# Genome-Wide Analysis of the Mads-Box Transcription Factor Family in *Solanum melongena*

**DOI:** 10.3390/ijms24010826

**Published:** 2023-01-03

**Authors:** Qi Chen, Jing Li, Fengjuan Yang

**Affiliations:** 1 State Key Laboratory of Crop Biology, College of Horticulture Science and Engineering, Shandong Agricultural University, Tai’an 271018, China; 2 Key Laboratory of Biology and Genetic Improvement of Horticultural Crop (Huang-Huai Region), Ministry of Agriculture and Rural Affairs, Tai’an 271018, China; 3 Shandong Collaborative Innovation Center for Fruit and Vegetable Production with High Quality and Efficiency, Tai’an 271018, China

**Keywords:** eggplant, MADS-box, genome-wide analysis, floral organ specification, anthocyanins biosynthesiss

## Abstract

The MADS-box transcription factors are known to be involved in several aspects of plant growth and development, especially in floral organ specification. However, little is known in eggplant. Here, 120 eggplant *MADS-box* genes were identified and categorized into type II (MIKCC and MIKC*) and type I (Mα, Mβ, and Mγ) subfamilies based on phylogenetic relationships. The exon number in type II SmMADS-box genes was greater than that in type I SmMADS-box genes, and the K-box domain was unique to type II MADS-box TFs. Gene duplication analysis revealed that segmental duplications were the sole contributor to the expansion of type II genes. *Cis*-elements of MYB binding sites related to flavonoid biosynthesis were identified in three SmMADS-box promoters. Flower tissue-specific expression profiles showed that 46, 44, 38, and 40 MADS-box genes were expressed in the stamens, stigmas, petals, and pedicels, respectively. In the flowers of *SmMYB113*-overexpression transgenic plants, the expression levels of 3 SmMADS-box genes were co-regulated in different tissues with the same pattern. Correlation and protein interaction predictive analysis revealed six SmMADS-box genes that might be involved in the *SmMYB113*-regulated anthocyanin biosynthesis pathway. This study will aid future studies aimed at functionally characterizing important members of the MADS-box gene family.

## 1. Introduction

In plants, MADS-box genes are one of the largest transcription factor (TF) families, and they play key roles in nearly every process related to plant growth and development [1,2,3]. The name MADS-box is derived from the initials of four TFs that were first discovered in this family: mini chromosome maintenance 1 (MCM1), agamous (AG), deficient (DEF), and serum response factor (SRF) [4]. MADS-box TFs have a highly conserved DNA-binding domain with approximately 60 amino acids at the N-terminus [5]. MADS-box TFs are divided into two distinct types according to their conserved protein domains and phylogenetic relationships: SRF-like (type I) and MEF2-like (type II) [6]. Type I proteins are composed of SRF-like domains and variable domains. Type I MADS-box genes can be further divided into three subgroups according to differences in their SRF-like domains: Mα, Mβ, and Mγ. Type II proteins are composed of a MEF2-like domain (M), intermediate domain (I), keratin-like domain (K), and a C-terminal domain. Type II proteins can be further subdivided into two subgroups, MIKC^C^ and MIKC^*^, based on structural differences between the I and K domains [7].

The type I MADS-box genes are also known as M-type genes. According to previous studies, only a few of the type I MADS-box genes have biological characteristics [8]. In *Arabidopsis*, PHE1 controls endosperm gene imprinting and seed development [9]. In rice, OSMADS78 and OSMADS79 regulate rice seed development [9]. Type II MADS-box genes, also known as MIKC-type genes, play more roles in plant growth and development than type I MADS-box genes. The first MADS-box gene found in plants was a type-II MADS-box gene involved in flower development [4]. The MADS-box genes involved in regulating the ABCDE model are mostly type II MADS-box genes [10]. Type II MADS-box genes regulate floral organ development as well as flowering time. For example, FLC TFs play key roles in regulating flowering time in *Arabidopsis*, and genetic variation in FLC activity can alter or eliminate the requirement of vernalization in different *Arabidopsis* ecotypes [11]. In addition, type II MADS-box genes can regulate seed and fruit development. In grapes, VvAGL11 regulates seed development, and the length of an extended single sequence repeat in the *VvAGL11* promoter region is inversely proportional to the degree of seed development [12,13]. In oil palm, SHELL regulates fruit morphology [14]. Recent studies have reported that MADS-box genes are involved in plant root development. In *Arabidopsis*, AGL12 regulates root meristem cell division and promotes the formation of root vascular tissue, which in turn regulates root structure and development [15].

MADS-box gene family members have been identified in many species. In *Arabidopsis*, 107 MADS-box gene family members have been identified, including 61 type I genes and 45 type II genes [16]. In rice, 76 MADS-box gene family members have been identified, including 32 type I genes and 44 type II genes [17]. In tomato, 107 MADS-box gene family members have been identified, including 53 type I genes and 54 type II genes [18]. In potatoes, 153 MADS-box gene family members have been identified, including 114 type I genes and 39 type II genes [19]. In cucumber, 44 MADS-box gene family members have been identified, including 10 type I genes and 33 type II genes [20].

Eggplant (*Solanum melongena* L.) is an economically important crop in the genus Solanum (family Solanaceae) [21]. Eggplant has a long history of cultivation in Asia, Africa, Europe, and other regions [22]. Eggplant is enriched in nutrients, especially anthocyanins [23,24], which can enhance eyesight and prevent cardiovascular disease. Both biotic and abiotic stresses can inhibit the development of floral organs, and anthocyanins play an important role in protecting plants from various stresses. So, increasing anthocyanin content is an important goal in eggplant breeding. Previous studies have shown that the MADS-box TF transparent testa 16 and 15 can regulate the accumulation of proanthocyanidins in the seed coat of *Arabidopsis* [25]. The MADS-box TF MdJa2 regulates the biosynthesis of anthocyanins and proanthocyanidins in red-fleshed apples through its role in the brassinosteroid signaling pathway [26]. No studies have examined the roles of MADS-box TFs in eggplant anthocyanin biosynthesis. Here, we studied eggplant MADS-box genes by conducting phylogenetic analysis, as well as analyses of the chromosomal locations of genes, gene structure, conserved motifs, and expression profiles. We also highlight the potential role of the MADS-box gene in flower development and anthocyanin biosynthesis in eggplant. Our findings provide new insights that will aid future studies aimed at clarifying the functions of the MADS-box gene in plants.

## 2. Results

### 2.1. Identification and Phylogenetic Analysis of SmMADS-Box Genes in Eggplant

**Figure 1:**
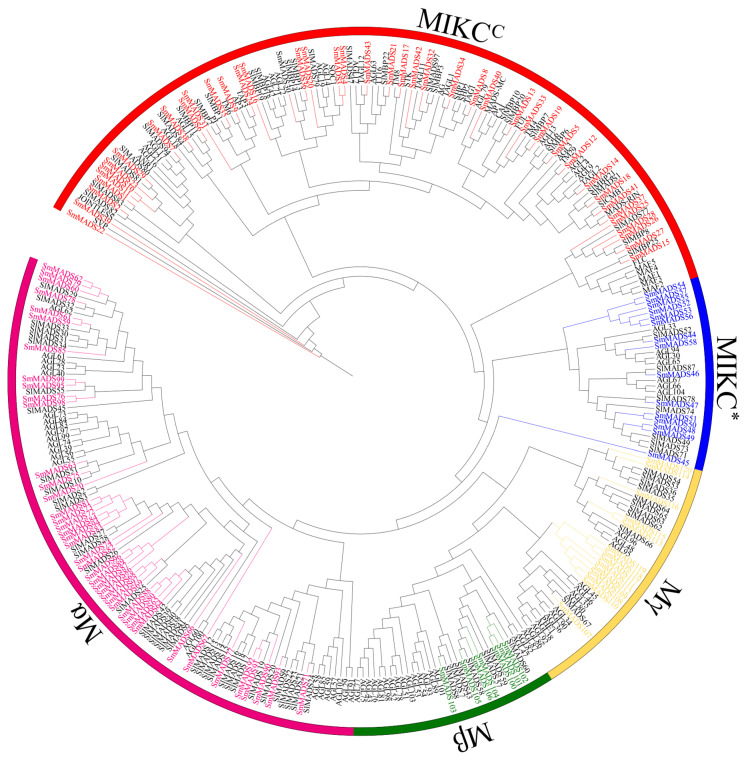
Phylogenetic analysis of MADS-box TFs from *Arabidopsis*, tomato, and eggplant. The MADS-box TFs from *Arabidopsis* and tomato are black. SmMADS-box genes are colored: red genes are MIKC^C^ type, blue genes are MIKC* type, purple genes are Mα type, green genes are Mβ type, and yellow genes are Mγ type.

Two bioinformatic approaches were used to identify MADS-box TFs in eggplant. A local BLASTP search with an e-value of 1 × 10^−3^ was performed using *Arabidopsis* MADS-box proteins as queries, which yielded 144 SmMADS-box candidate genes. A total of 151 coding sequences were identified based on recently published functional annotations (Pfam domain) in the SGN eggplant genome. These candidate genes were submitted to the NCBI CDD and Pfam databases to confirm the presence of the MADS-box domain. After removing redundant sequences, a total of 120 SmMADS-box genes were identified in eggplant. These genes were named SmMADS1 to 120 according to their chromosomal location and subfamily affiliation (Figure 1). The SGN site/gene name, amino acid sequence length, molecular weight, and pI of the 120 MADS-box genes are listed in Table A1. The predicted amino acid sequence lengths of the 120 SmMADS-box proteins ranged from 53 (SmMADS45) to 591 (SmMADS58), and the relative molecular weights ranged from 6018.15 Da (SmMADS45) to 65,950.08 Da (SmMADS58). pI ranged from 4.54 (SmMADS58) to 11.39 (SmMADS29). Most of the type II genes contained multiple exons, whereas the type I genes generally contained only one exon.

The protein sequences of 120 SmMADS-box TFs, 100 SlMADS-box TFs, and 102 AtMADS-box TFs were pre-matched using ClustalX (1.83), and the results were used to construct NJ trees with MAGE 11 with 1000 bootstrap replicates. According to the phylogenetic relationships of SmMADS-box proteins in *Arabidopsis*, tomato, and eggplant, the 120 SmMADS-box TFs were classified into 62 type I and 58 type II subfamilies (Figure 1). The 62 type I SmMADS-box TFs were divided into 40 Mα, 6 Mβ, and 14 Mγ genes. The 58 type II MADS-box TFs were further divided into 44 MIKC^C^ and 14 MIKC^*^ type genes.

### 2.2. Gene Structure and Conserved Motif Analysis of the 120 SmMADS-Box Tfs

**Figure 2:**
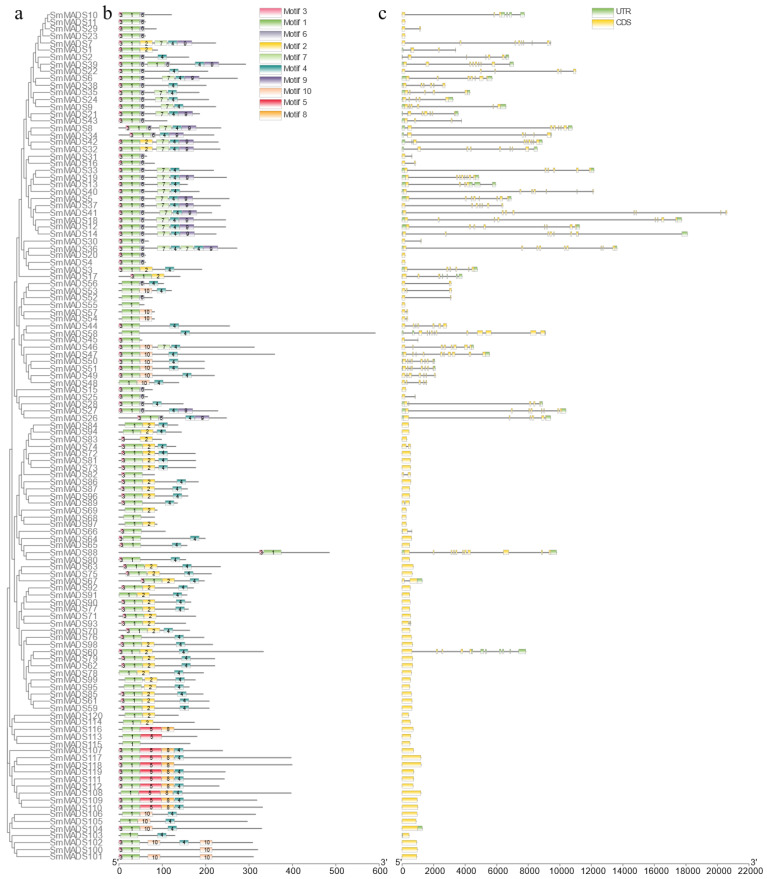
Phylogenetic relationships, conserved motifs, and gene structures of SmMADS-box genes. (**a**) The phylogenetic relationships of the 120 SmMADS-box TFs; 1000 bootstrap replicates were used to evaluate node support. (**b**) Conserved motifs from 120 SmMADS-box genes were analyzed using MEME. Rounded rectangles with different colors indicate different motifs. (**c**) The gene structures of the SmMADS-box genes were analyzed using TBtools. Exons and UTRs are colored in green and yellow boxes, respectively, and black lines represent introns.

**Figure A1:**
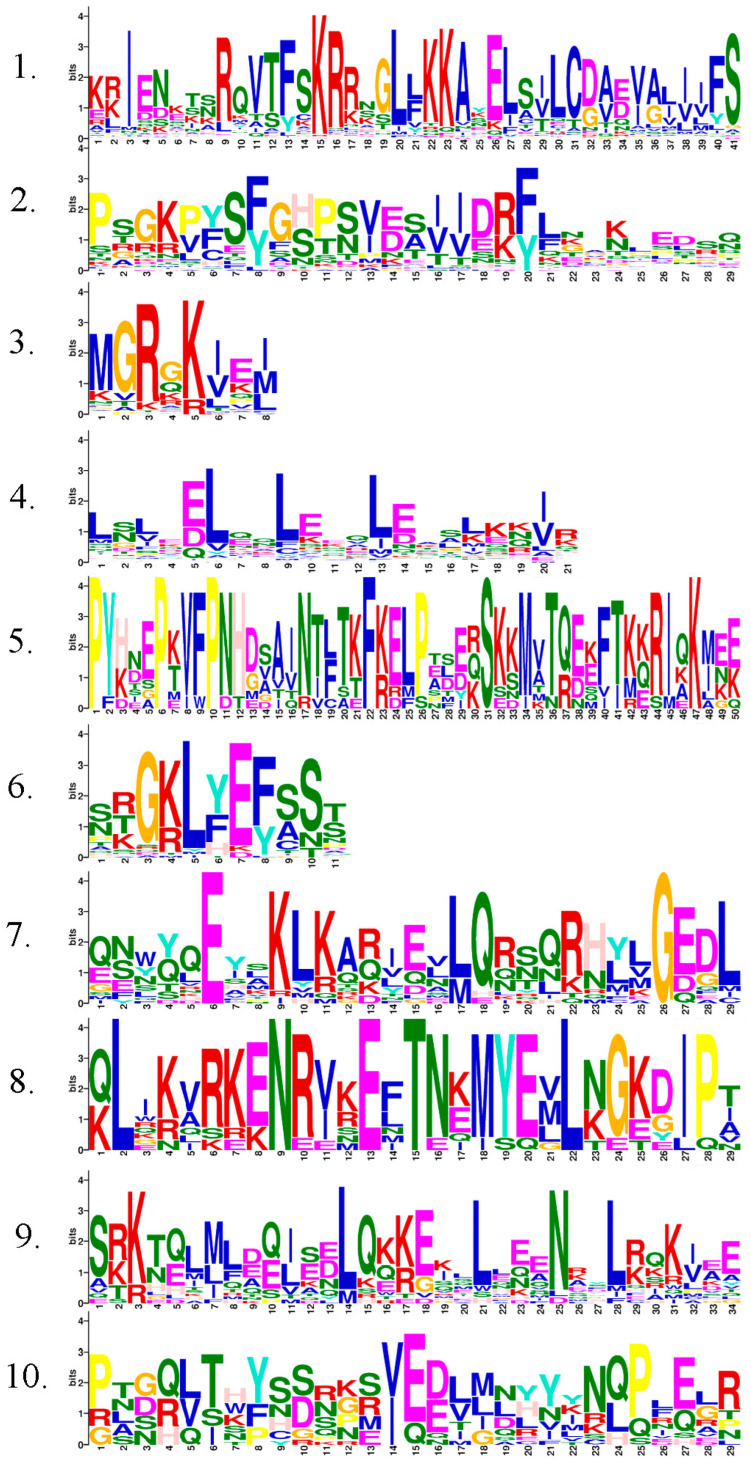
Multilevel consensus sequence among MADS-box genes in eggplant was identified by MEME.

To further analyze the composition of the 120 SmMADS-box TFs, the conserved motifs were analyzed using MEME online software. A total of 10 conserved motifs were identified, and these were referred to as motifs 110 (Figure 2b and Figure A1). The SmMADS-box domain consists of Motifs 1, 2, 3, and 6. Motif 7 contains the K domains, which play an important role in protein-protein interactions between SmMADS-box proteins; this domain is only present in type II SmMADS-box proteins (Figure 2b). Most of the type II genes contain multiple exons, and type I genes generally contain only one exon (Figure 2c).

### 2.3. Chromosomal Location, Gene Duplication, and Homology Analysis of the 120 SmMADS-Box Genes

**Figure 3:**
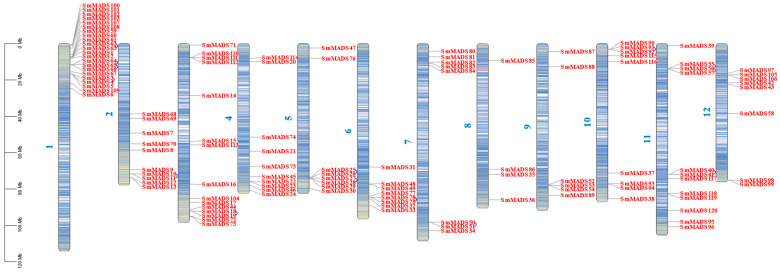
Physical map of 120 SmMADS-box genes in 12 chromosomes. The blue arcs in the graph correspond to genes that have undergone tandem duplications.

TBtools software was used to map the physical position of the 120 SmMADS-box genes on the 12 chromosomes of eggplant; this information could aid future studies of the functions of SmMADS-box genes in eggplant. Based on the chromosomal locations of the 120 SmMADS-box genes, the three chromosomes with the most *SmMADS-box* genes are Chr01 (22 genes), Chr03 (15 genes), and Chr11 (12 genes) (Figure 3).

**Figure 4:**
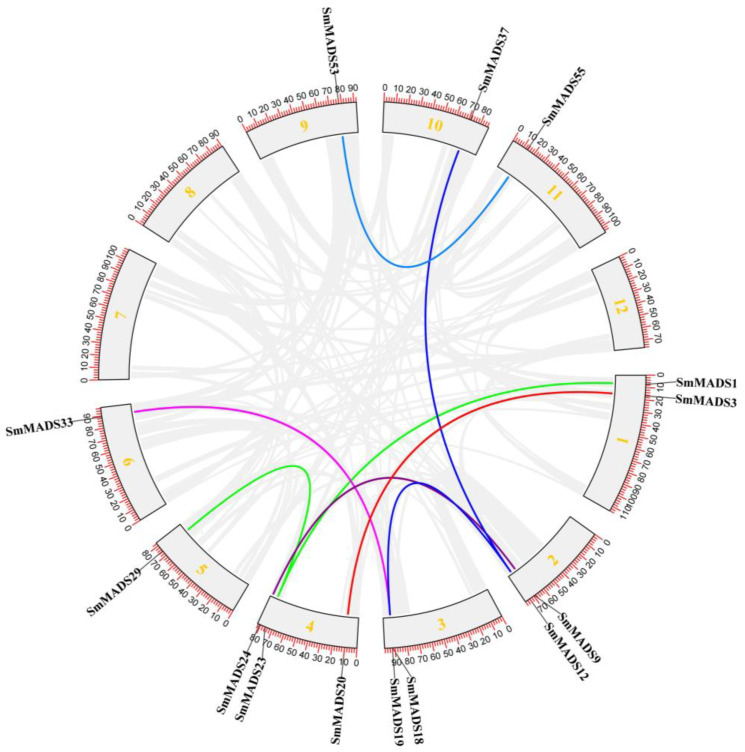
Segmental duplication of 120 SmMADS-box genes in 12 chromosomes. Genes linked with a line are pairs of segmentally duplicated genes.

Gene duplication events in the SmMADS-box gene family were analyzed. A total of 31.6% (38 of 120) of *SmMADS-box* genes were derived from gene duplications (Figure 3 and Figure 4). Tandemly duplicated genes were mainly located on chromosome 1 and chromosome 11 and accounted for approximately 42.9% of tandemly duplicated genes. Among them, 17 tandemly duplicated genes were type II genes; 11 of these tandemly duplicated genes were type I genes (Figure 3). Among the 120 *SmMADS-box* genes, fragment repeats accounted for only 11.7% of all genes, and 100% of these genes were type II genes; two fragment repeats were observed in *SmMADS12* and *SmMADS23* (Figure 4). These findings indicate that fragment repeats and tandem repeats have played an important role in the expansion of type II genes, and tandem duplication has played a key role in the expansion of type I genes.

**Figure 5:**
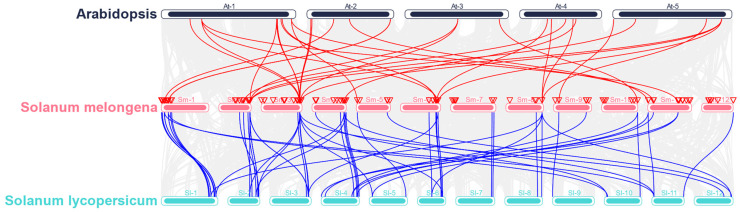
Syntenic relationships between homologous SmMADS-box genes of tomato and other species. The MADS-box gene pairs between different species are highlighted with different colored lines.

To further identify the homologs of the 120 SmMADS-box genes between eggplant and other plant species, the synteny of *Arabidopsis* and *Solanum lycopersicum* (tomato) plants with eggplant was analyzed by MCScanX. The results showed that 20 SmMADS-box genes were collinear with Arabidopsis genes and that 42 SmMADS-box genes were collinear with tomato genes (Figure 5). *SmMADS13*, *SmMADS17*, *SmMADS36*, and *SmMADS44* were related to at least three pairs of homologs. We speculate that these genes might play important roles in the evolution of the MADS-box gene family. These findings indicate that MADS-box genes in Arabidopsis, eggplant, and tomato exhibit strong synteny.

### 2.4. Cis-Regulatory Elements in the Promoters of the 120 SmMADS-Box Genes

**Figure 6:**
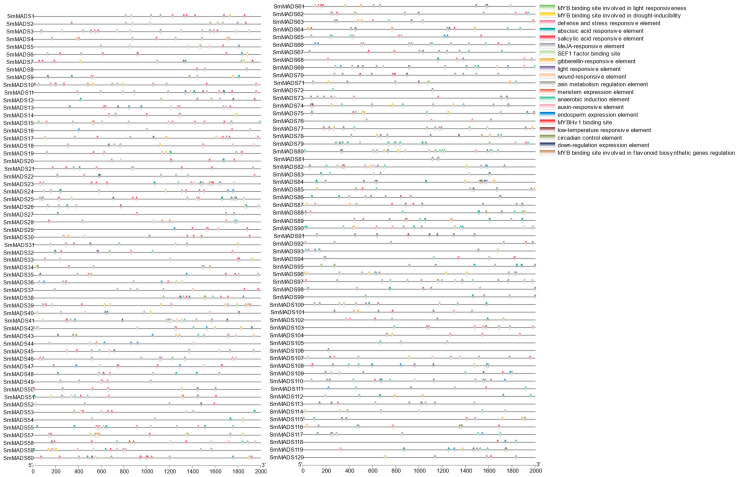
*Cis*-regulatory elements in the 2-kb upstream region of 120 SmMADS-box coding sequences. Rounded rectangles with different colors indicate different *cis*-acting elements.

To clarify the biological pathways in which the 120 SmMADS-box genes participate in eggplant, cis-regulatory elements in the promoter sequences were analyzed. Four types of cis-elements were detected, including light-responsive, stress-responsive, hormone-responsive, and binding sites (Figure 6). The light-responsive elements were found in the promoters of 103 SmMADS-box genes, the hormone-responsive elements were found in the promoters of 116 SmMADS-box genes, and MYB binding sites were found in the promoters of 66 MADS-box genes. MYB-binding sites involved in flavonoid biosynthesis were found in SmMADS6, SmMADS58, and SmMADS107 (Figure 5).

### 2.5. Flower Tissue-Specific Expression Patterns of SmMADS-Box Genes

**Figure 7:**
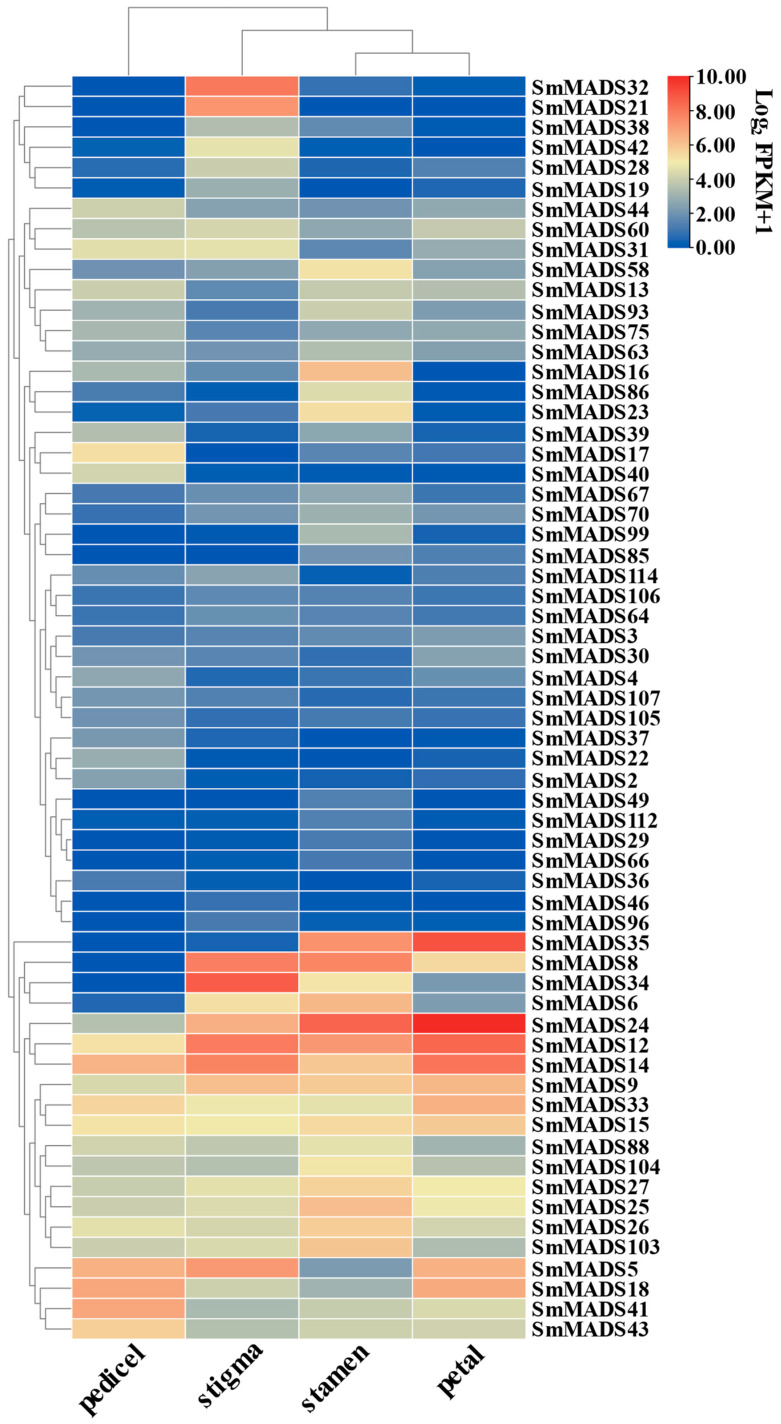
The expression levels of SmMADS-box genes in different floral organs. Redder colors indicate higher expression levels, and bluer colors indicate lower expression levels.

To explore the functions of *SmMADS-box* genes in flower development, the expression levels of *SmMADS-box* genes were characterized using the Log2 FKPM (fragments per kilobase transcript per million mapped fragments) method, and the data were normalized using the zero-to-one method according to RNA-seq data from stamens, stigmas, petals, and pedicels. The number of SmMADS-box genes expressed in different organs differed; specifically, 46, 44, 38, and 40 SmMADS-box genes were detected in stamens, stigmas, petals, and pedicels, respectively. The eggplant petals of the sequenced varieties were lavender, and no anthocyanin accumulation was detected in other floral organs; thus, *SmMADS-box* genes with higher expression levels in petals might be involved in anthocyanin biosynthesis. *SmMADS3*, *SmMADS9*, *SmMADS12*, *SmMADS14*, *SmMADS15*, *SmMADS18*, *SmMADS24*, *SmMADS30*, *SmMADS33*, and *SmMADS35* were highly expressed in petals, suggesting that these *SmMADS-box* genes might be involved in anthocyanin biosynthesis (Figure 7).

### 2.6. Correlation Analysis of Eggplant SmMADS-Box Genes and SMMYB113

MYB binding sites involved in regulating flavonoid biosynthesis genes were identified in the promoter sequences of *SmMADS6*, *SmMADS58*, and *SmMADS107* (Figure 6). *SmMYB113* is an important regulator of anthocyanin biosynthesis [27,28]. The anthocyanin content was significantly higher in whole *SmMYB113*-overexpressing plants than in wild-type (WT) plants; the same difference was also observed in the stamens, petals, and pedicels. However, the flower abscission rate of *SmMYB113*-overexpression plants was significantly higher than that of WT plants. Therefore, we speculated that *SmMYB113* might play a role in floral development.

**Figure 8:**
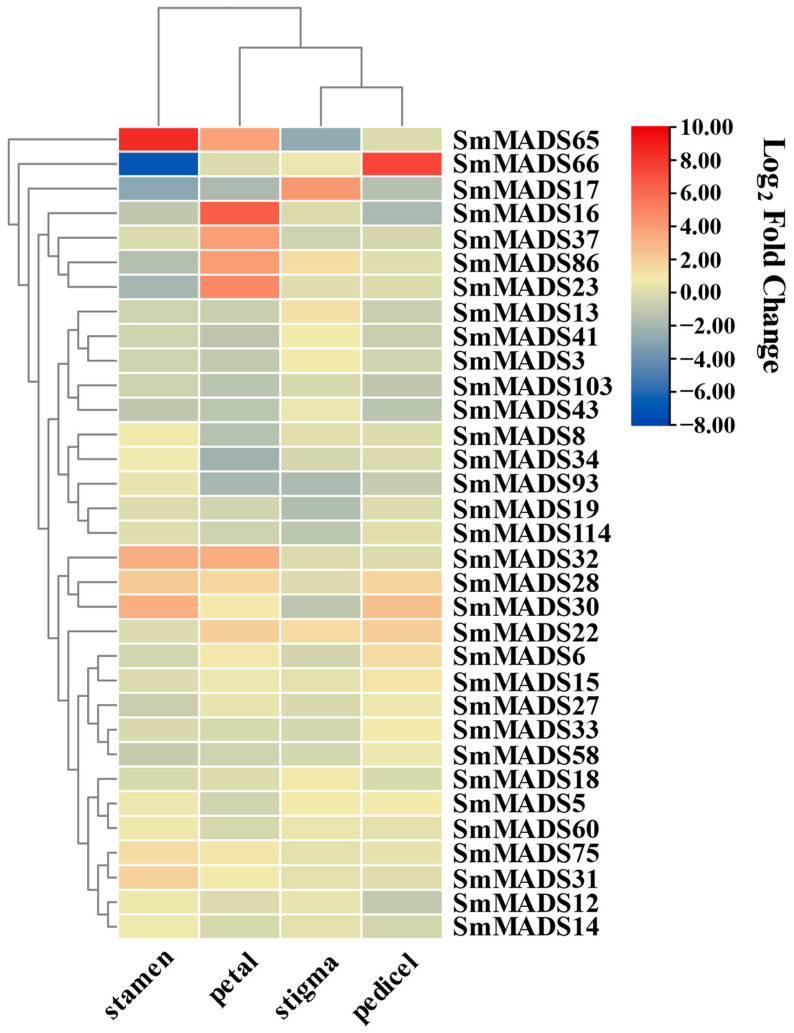
The differential expression of SmMADS-box genes in *SmMYB113*-overexpressed plants compared with WT plants. Redder colors indicate higher expression levels, and bluer colors indicate lower expression levels.

**Figure A2:**
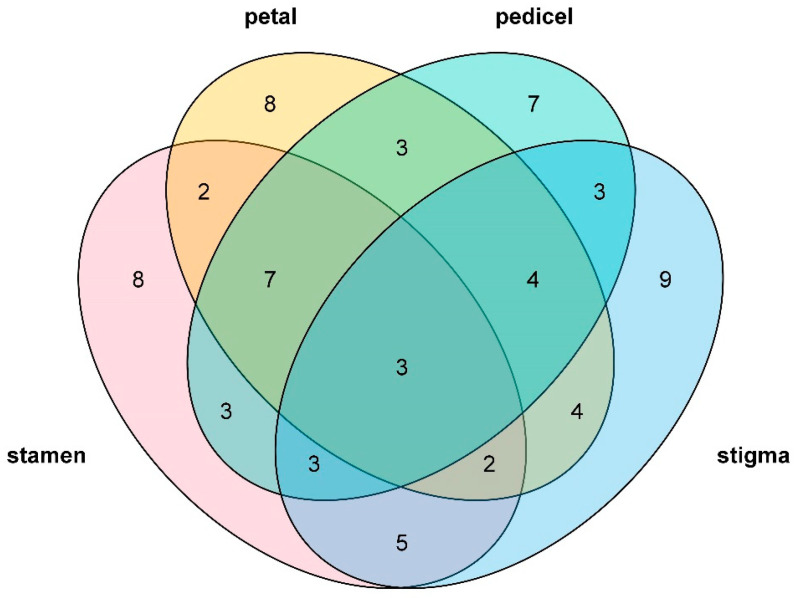
Veen diagram analysis of DEGs.

To explore the relationship between SmMADS-box genes and *SmMYB113*, as well as their effects on floral development and anthocyanin biosynthesis, the expression level of SmMADS-box genes in *SmMYB113*-overexpressed flowers and the relationships between SmMADS-box genes and anthocyanin biosynthesis were analyzed. According to RNA-seq data from stamens, stigmas, petals, and pedicels, the expression levels of 33 SmMADS-box genes varied among floral organs; these included 29 genes in stamens, 31 genes in stigmas, 30 genes in petals, and 27 genes in pedicels. Further analysis revealed that the expression of 14 genes was up-regulated and the expression of 15 genes was down-regulated in the stamens. In the stigma, the expression of 18 genes and 13 genes was up-regulated and down-regulated, respectively. In the petals, the expression of 14 genes and 16 genes was up-regulated and down-regulated, respectively. In the pedicel, the expression of 15 genes and 12 genes was up-regulated and down-regulated, respectively (Figure 8). Further analysis revealed that the expression of three SmMADS-box genes might be regulated by SmMYB113, suggesting that SmMYB113 may be involved in flower organ formation by regulating the expression of SmMADS-box genes (Figure A2).

**Figure 9:**
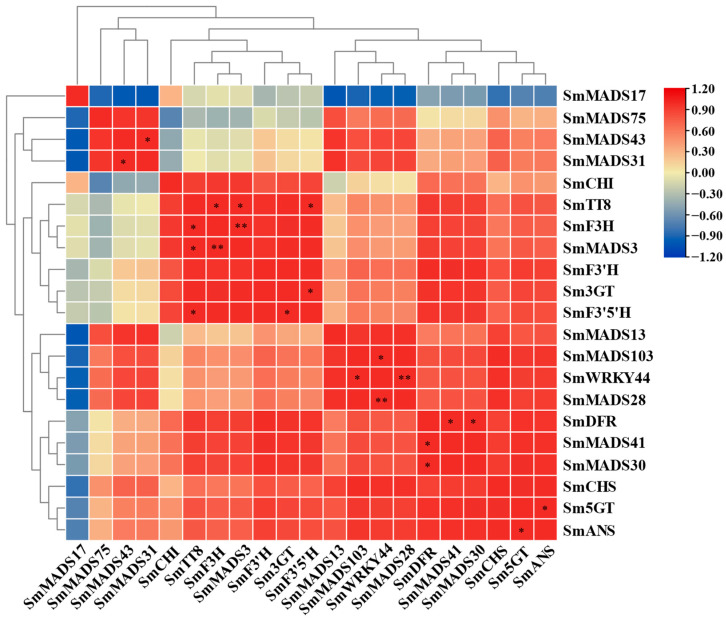
The expression correlation profiles of SmMADS-box genes in different floral tissues of SmMYB113-overexpressing plants and WT plants. Different colors indicate different correlations, with the highest in red and the lowest in blue. * indicates a significant level (*p* < 0.05) and ** indicates highly significant correlations (*p* < 0.01).

Given that the anthocyanin content was higher in the stamens, petals, and pedicels of *SmMYB113*-overexpression plants than in WT plants, and no significant differences in the anthocyanin content were observed in the stigmas of SmMYB113-overexpression plants and WT plants, we examined SmMADS-box genes that were differentially expressed in the stamens, petals, and pedicels. The expression of four SmMADS-box genes was co-upregulated by SmMYB113, and the expression of six SmMADS-box genes was co-downregulated by SmMYB113 (Figure 9). Correlations between the 10 SmMADS-box genes and 11 anthocyanin biosynthesis-related genes were analyzed using SPSS. The expression of *SmMADS3* was significantly associated with the expression of *SmF3H* and *SmTT8*, the expression of *SmMADS30* and *SmMADS41* was significantly associated with the expression of *SmDFR*, and the expression of *SmMADS28* and *SmMADS103* was significantly associated with the expression of *SmWRKY44*. The above findings indicate that *SmMADS3*, *SmMADS28*, *SmMADS30*, *SmMADS41,* and *SmMADS103* might be involved in the SmMYB113-regulated anthocyanin biosynthesis pathway (Figure 9).

### 2.7. Protein Interaction Predictive Analysis of SmMADS-Box Tfs and Anthocyanin Biosynthesis-Related Genes

**Figure 10:**
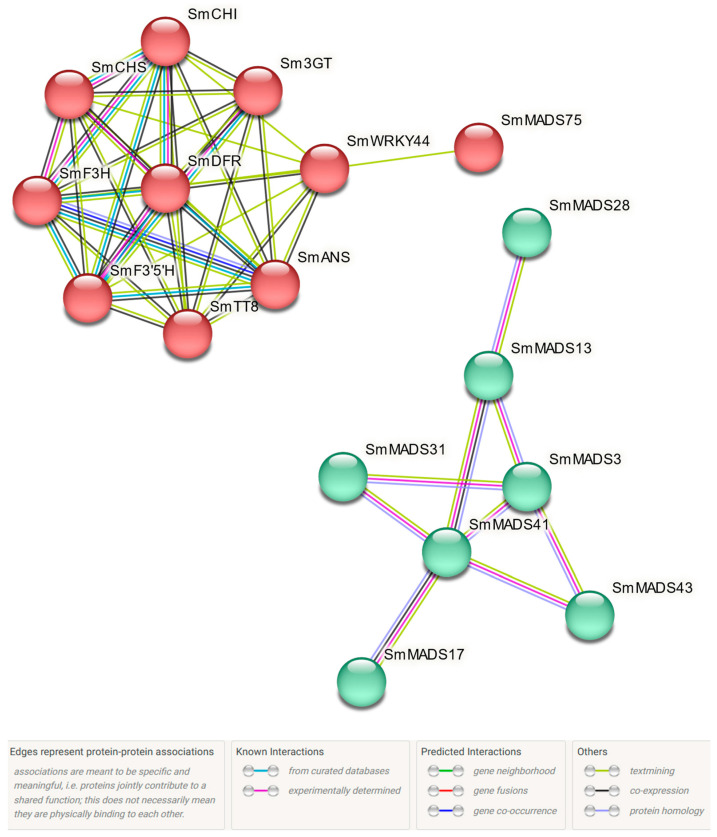
Protein-protein interaction network of SmMADS TFs. Edges indicate protein-protein associations.

To further analyze the role of these SmMADS-box TFs and determine whether they are involved in anthocyanin biosynthesis according to their expression patterns, STRING software was used to predict interaction networks between 10 SmMADS-box genes regulated by SmMYB113 and anthocyanin biosynthesis-related genes in the transcriptomes of different floral organs in eggplant. Anthocyanin biosynthesis-related genes were closely linked internally; anthocyanin biosynthesis-related genes and SmMADS-box genes were also linked. *SmMADS75* was linked to *SmWRKY44* (Figure 10), suggesting that this SmMADS-box gene might play a role in anthocyanin biosynthesis by affecting the expression of genes encoding TFs. In addition, SmMADS-box genes are also closely linked to each other, and it is speculated that SmMADS-box genes may participate in anthocyanin biosynthesis and play other roles through the same pathway. The relationships and functions within SmMADS-box genes, within anthocyanin biosynthesis-related genes, and between SmMADS-box and anthocyanin biosynthesis-related genes deserve our in-depth study and attention.

### 2.8. Effect of SMMYB113 Overexpression on the Expression Level of Six Selected Smmadx-Box Genes

**Figure 11:**
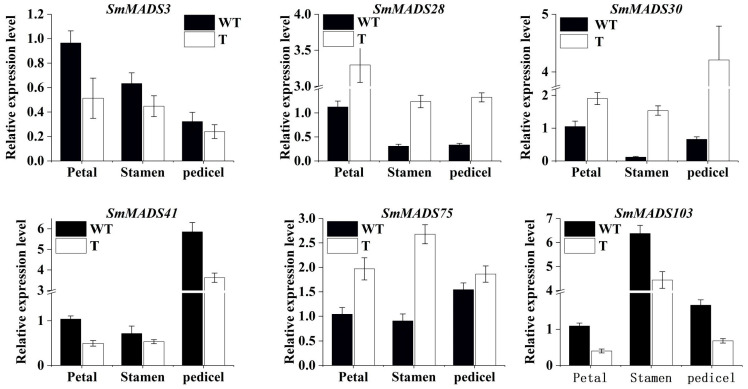
The expression analysis of SmMADS3, SmMADS28, SmMADS30, SmMADS41, SmMADS75, and SmMADS103 in wild-type and SmMYB113 overexpression eggplant. The expression of each gene in the petal of WT was set to 1. The qRT-PCR was calculated using 2^−(ΔΔCT)^ method. The data are shown as ± SE (*n* = 3).

Six SmMADS-box genes were identified as potentially involved in anthocyanin biosynthesis according to the expression profiles of SmMADS-box genes and protein interaction network analysis. qRT-PCR was conducted to validate the RNA-seq data (Figure 11). The expression patterns of the six SmMADS-box genes were basically consistent with the RNA-seq data, suggesting that the RNA-seq data were reliable.

## 3. Discussion

MADS-box genes regulate a variety of biological processes in plants, including vegetative and reproductive growth; they also play key roles in inflorescence, flower, and fruit development [29,30,31,32]. The publication of a greater number of high-quality genomes has aided genome-wide analyses. Several MADS-box gene family members have been identified in various plants, including *Arabidopsis* (107) [16], rice (75) [17], tomato (131) [18], potato (153) [19], bread wheat (300) [33], cucumber (43) [20], *Brassica rapa* (167) [34], radish (144) [35], apple (146) [36], sesame (57) [37], chrysanthemum (108) [38], and watermelon (39) [39]. In this study, 120 SmMADS-box genes were identified in eggplant. They were divided into type II (MIKC^C^ and MIKC^*^) and type I (Mα, Mβ, and Mγ) subfamilies according to their phylogenetic relationships. The number of type II members is generally greater than the number of type I members [16,17,33,34,35,36,37,38]; in this study, the number of type I members was greater than the number of type II members in eggplant. In addition, the structure of type II SmMADS-box genes was more complex than that of type I genes because type II genes contained more exons than type I genes. Protein motif analysis showed that MADS-box genes all contained a conserved MADS domain, and the K-box domain was unique to type II MADS-box genes, suggesting that type II genes may have more diverse functions. These results are similar to the findings of previous studies in species such as Arabidopsis, rice, tomato, and potato. [16,17,18,19]. Gene duplication analysis revealed that tandem duplications have contributed to expansions of type I and type II genes in eggplant and segmental duplications have contributed to the expansion of type II genes. However, in *Arabidopsis* and potato, tandem and segmental duplications have both contributed to expansions of type I and type II genes [16,19]. In cucumber, only tandem duplications have been documented [20]. This suggests that gene duplication plays different roles in different species. 20 pairs of collinear MADS-box genes between eggplant and *Arabidopsis* and 42 pairs of collinear MADS-box genes between eggplant and tomato were further identified. The number of homologous events between eggplant and tomato was much larger than that between eggplant and *Arabidopsis*, which is consistent with the small evolutionary distance between eggplant and tomato.

The effects of MADS-box family members on floral organ development have been thoroughly investigated by previous authors in various species. In *Arabidopsis*, SOC1-like genes AGL42, AGL71, and AGL72 promote flowering in the shoot apical and axillary meristems [40]. Another study has shown that AGL42 regulates flower senescence in *Arabidopsis* [41]. The AGL62 MADS domain protein regulates cellularization during endosperm development in *Arabidopsis* [42]. In tomato, the MADS-box family members jointless (J), macrocaylyx (MC), and SLMBP21 form a complex that regulates the development of pedicel-free cells and thus flower abscission [43]. In bananas, *MuMADS1* is involved in ethylene-induced fruit ripening [44]. In Japanese gentian, SEP proteins in control STK, AG, SHP1, and SHP2 form a multimeric complex to control normal ovule development [45]. Here, 46, 44, 38, and 40 MADS-box genes were highly expressed in stamens, stigmas, petals, and pedicels, respectively. *SmMADS21* and *SmMADS32* were highly expressed in the stigma, *SmMADS86* was expressed in the stamen, *SmMADS17* was highly expressed in the pedicel, and *SmMADS24* was highly expressed in the petal, indicating that these genes might have specific functions in the structures in which they were most highly expressed. These findings will help to study the function of SmMADS-box genes in flower development. SmMYB113 is an important regulator of anthocyanin biosynthesis [27,28]. However, the flower abscission rate of *SmMYB113*-overexpression plants was significantly higher than that of WT plants. According to the RNA-seq data, the expression of *SmMADS31*, *SmMADS75*, and *SmMADS103* was regulated by SmMYB113, and these genes are homologs of *AGL42*, *AGL62*, and *AGL103* in *Arabidopsis*, respectively. Therefore, we speculated that SmMYB113 might be involved in regulating flower and leaf senescence, endosperm development, and the polar transport of growth hormone [46].

In some plants, MADS-box family genes are closely related to the differentiation of anthocyanins or proanthocyanidins, and these MADS-box genes that affect anthocyanin synthesis and accumulation are all type II genes. In orchids, OAP3, OAGL2, and OPI form a complex to regulate anthocyanin accumulation [47,48]. In kiwifruit, SVP3 regulates the transcription of the R2R3-MYB regulator *MYB110a* and the anthocyanin biosynthesis structural gene *F3GT1*, which affects petal pigmentation [49]. In Cineraria (*Senecio cruentus*), ScAG directly represses the transcription of *ScF3H1*, and *ScAGL11* directly represses the expression of *ScDFR3*, respectively, affecting the biosynthesis of anthocyanins [50]. In this study, a total of six MADS-box genes that might be involved in anthocyanin biosynthesis were identified, including the type II MADS-box genes *SmMADS3*, *SmMADS28*, *SmMADS30*, and *SmMADS41*, and the type I MADS-box genes *SmMADS75* and *SmMADS103*. These findings provide information that will aid future studies of the role of the SmMADS-box genes in anthocyanin biosynthesis. In addition, SmMADS-box genes were found to be associated with *SmWRKY44* in the expression pattern, correlation, and interaction network analysis; *SmWRKY44* might play an important role in regulating anthocyanin biosynthesis by modulating the expression of SmMADS-box genes.

## 4. Materials and Methods

### 4.1. Identification of MADS-Box Genes in Eggplant

First, *Arabidopsis* protein sequences were used as queries in local BLASTP searches [16] with an e-value of 1 × 10^−3^ to identify predicted eggplant MADS-box proteins [51]. Functional annotations were filtered using the Protein family database (Pfam) identifiers of the MADS and K domains (PF00319 and PF01486, respectively) [52]. All putative MADS-box sequences were collected, and redundant sequences were manually removed; the remaining candidate MADS-box sequences were subjected to NCBI Conserved Domain (CD) searches (https://www.ncbi.nlm.nih.gov/Structure/cdd/wrpsb.cgi, accessed on 30 September 2022) and Pfam Batch searches (https://pfam.xfam.org/, accessed on 30 September 2022) to confirm the existence of MADS-box domains. Next, we obtained DNA sequences based on their amino acid sequences from the SGN database. The physical and chemical parameters of SmMADS-box proteins, including the number of amino acids, molecular weight, and theoretical pI, were predicted using Expasy ProtParam (https://web.expasy.org/protparam/, accessed on 30 September 2022).

### 4.2. Phylogenetic, Conserved Motifs, and Gene Structure Analyses of MADS-Box Genes in Eggplant

Eggplant, tomato [18], and *Arabidopsis* [16] MADS-box protein sequences were aligned using ClustalX with default parameters, and the result was pruned using the Trimmer function in TBtools. Phylogenetic trees were generated using the neighbor-joining (NJ) method in MEGA11 with 1000 bootstrap replicates to evaluate node support [53]. The Interactive Tree of Life (iTOL) was used to visualize the phylogenetic tree (https://itol.embl.de/, accessed on 30 September 2022) [54,55].

The MEME Suite v5.4.1 online tool (http://meme-suite.org/meme/tools/meme, accessed on 21 October 2022) was used to predict the SmMADS-box conserved motifs [56]. The maximum number of motifs was set to 10, and other parameters were set to their default values. The structure of SmMADS-box genes and figures of gene structure were generated using Gene Structure View in TBtools.

### 4.3. Chromosomal Location, Gene Duplication, and Homology Analysiss

Using the GFF function in TBtools software, the GFF3 file downloaded from SGN (http://solgenomics.net/, accessed on 16 September 2022) was used to identify the position of MADS-box genes in the physical map of the eggplant genome [57]. Eggplant MADS-box genes were named based on the position information obtained from SGN. According to the nomenclature method used in rice, the eggplant MADS-box genes were named SmMADS1 to SmMADS120 following the order of MIKC^C^, MIKC^*^, Mα, Mβ, and Mγ. Using the genome file and GFF3 file downloaded from SGN and Ensembl Plants, the one-step MCScanX function in TBtools was used to analyze the collinearity of MADS-box genes, and the advanced Circos function in TBtools was used to draw the collinearity circle diagram [57,58]. The syntenic relationship between orthologous SmMADS-box genes of eggplant and other selected species was determined using the Dual Synteny Plotter tool in TBtools software. Tandemly duplicated genes were determined in SGN with the criterion that no more than one gene be shared between two genes with high homology (>50%).

### 4.4. Cis-Element Analysis

*Cis*-element analysis was conducted using 2.0-kb sequences upstream of the translational start site of *SmMADS-box* genes with the online PlantCARE tool (https://bioinformatics.psb.ugent.be/webtools/plantcare/html/, accessed on 3 October 2022).

### 4.5. Expression Pattern and Correlation Analysis

The expression patterns of MADS-box genes in eggplant were analyzed using the RNA-seq data. SPSS statistics was used to conduct correlation analysis, and TBtools was used to make expression heatmaps and correlation heatmaps [57].

### 4.6. Interaction Network

The interaction network was built using STRING (functional protein association networks, Available online: https://string-db.org/, accessed on 15 December 2022) software through searches of multiple protein sequences [59].

### 4.7. Total RNA Extraction and qPCR Analysis

Total RNA was extracted from sepals, petals, stamens, and stigmas using the Trizol method for each tissue; there were three biological replicates for each sample. First-strand cDNA was synthesized using TransScript@ One-Step gDNA Removal and cDNA Synthesis SuperMix (TRANS) reagents. qRT-PCR was performed in a 7500 Real-Time PCR System using TB Green^®^ Premix Ex TaqTM (Tli RNaseH Plus) (Takara) reagents. The 2^−(ΔΔCT)^ method was used to calculate the relative expression levels of SmMADS-box genes [60]. The SmActin gene was used to normalize the qRT-PCR data. The primers are listed in Table A2.

## 5. Conclusions

In this study, a total of 120 SmMADS-box genes were identified, and these were classified into two types: type I (Mα, Mβ, Mγ) and type II (MIKC^C^, MIKC^*^) genes. Both types of MADS-box genes contain a conserved MADS structural domain, and the K-box structural domain is unique to type II MADS-box genes. Fragment duplication and tandem duplication have played an important role in the expansion of type II genes, and tandem duplication has played an important role in the expansion of type I genes. According to RNA-seq data, a total of 62 SmMADS-box genes were found to potentially be involved in floral organ development, and six SmMADS-box genes were potentially involved in the *SmMYB113*-regulated anthocyanin biosynthesis pathway. These findings indicate that MADS-box genes have many potential functions that merit further study. The large amounts of information on eggplant MADS-box gene family members obtained in this study enhance our understanding of the structure-function relationships among eggplant MADS-box gene family members. Our results will aid future studies aimed at clarifying the functions of eggplant MADS-box gene family members.

## Data Availability

Not applicable.

## Appendix A

**Table A1:** The detailed information of eggplant MADS-box gene family.

Gene Name	Gene Locus	Protein			Exon	Type	Subfamily
		Length (aa)	MW (Da)	pI			
SmMADS1	SMEL4.1_01g007770.1	89	10,077.78	10.14	3	Type II	MIKC^C^
SmMADS2	SMEL4.1_01g008380.1	161	18,252.78	7.84	5	Type II	MIKC^C^
SmMADS3	SMEL4.1_01g016420.1	191	21,929.28	9.13	6	Type II	MIKC^C^
SmMADS4	SMEL4.1_01g016430.1	61	6963.16	11.29	1	Type II	MIKC^C^
SmMADS5	SMEL4.1_01g016450.1	254	28,691.55	8.73	8	Type II	MIKC^C^
SmMADS6	SMEL4.1_01g022190.1	273	30,804.84	5.88	7	Type II	MIKC^C^
SmMADS7	SMEL4.1_02g006940.1	223	25,119.47	9.00	9	Type II	MIKC^C^
SmMADS8	SMEL4.1_02g011710.1	235	27,308.08	9.43	6	Type II	MIKC^C^
SmMADS9	SMEL4.1_02g021760.1	223	25,814.66	9.70	6	Type II	MIKC^C^
SmMADS10	SMEL4.1_02g024180.1	121	13,446.38	10.30	3	Type II	MIKC^C^
SmMADS11	SMEL4.1_02g024190.1	61	6888.07	10.65	1	Type II	MIKC^C^
SmMADS12	SMEL4.1_02g026490.1	125	28,475.31	8.25	8	Type II	MIKC^C^
SmMADS13	SMEL4.1_02g026500.1	158	18,427.18	8.83	4	Type II	MIKC^C^
SmMADS14	SMEL4.1_03g008110.1	224	25,730.65	9.80	7	Type II	MIKC^C^
SmMADS15	SMEL4.1_03g010360.1	77	8983.52	10.69	1	Type II	MIKC^C^
SmMADS16	SMEL4.1_03g015990.1	82	9441.02	10.02	2	Type II	MIKC^C^
SmMADS17	SMEL4.1_03g022770.1	141	16,105.54	9.98	3	Type II	MIKC^C^
SmMADS18	SMEL4.1_03g027790.1	246	28,209.90	8.94	8	Type II	MIKC^C^
SmMADS19	SMEL4.1_03g027800.1	248	28,728.36	9.10	8	Type II	MIKC^C^
SmMADS20	SMEL4.1_04g005970.1	61	7065.29	10.65	1	Type II	MIKC^C^
SmMADS21	SMEL4.1_04g011260.1	186	21,740.04	8.81	6	Type II	MIKC^C^
SmMADS22	SMEL4.1_04g017530.1	205	23,042.58	8.43	6	Type II	MIKC^C^
SmMADS23	SMEL4.1_04g017760.1	61	6892.02	10.43	1	Type II	MIKC^C^
SmMADS24	SMEL4.1_04g021980.1	207	23,958.04	8.91	5	Type II	MIKC^C^
SmMADS25	SMEL4.1_05g017100.1	66	7668.98	10.62	2	Type II	MIKC^C^
SmMADS26	SMEL4.1_05g017110.1	248	28,275.08	8.45	6	Type II	MIKC^C^
SmMADS27	SMEL4.1_05g017480.1	228	26,042.78	7.13	8	Type II	MIKC^C^
SmMADS28	SMEL4.1_05g017490.1	148	16,831.34	6.91	5	Type II	MIKC^C^
SmMADS29	SMEL4.1_05g017760.1	86	9562.26	11.39	2	Type II	MIKC^C^
SmMADS30	SMEL4.1_05g022920.1	68	7793.10	10.28	2	Type II	MIKC^C^
SmMADS31	SMEL4.1_06g010960.1	64	7347.62	10.01	2	Type II	MIKC^C^
SmMADS32	SMEL4.1_06g018550.1	233	27,010.92	9.31	7	Type II	MIKC^C^
SmMADS33	SMEL4.1_06g022050.1	218	25,088.34	8.88	7	Type II	MIKC^C^
SmMADS34	SMEL4.1_07g020500.1	219	24,525.82	8.70	5	Type II	MIKC^C^
SmMADS35	SMEL4.1_08g015360.1	185	21,477.57	9.21	5	Type II	MIKC^C^
SmMADS36	SMEL4.1_08g022290.1	272	31,277.04	9.36	9	Type II	MIKC^C^
SmMADS37	SMEL4.1_10g015470.1	234	26,809.40	7.76	7	Type II	MIKC^C^
SmMADS38	SMEL4.1_10g025220.1	201	22,745.80	6.55	6	Type II	MIKC^C^
SmMADS39	SMEL4.1_11g000980.1	292	33,368.72	8.72	8	Type II	MIKC^C^
SmMADS40	SMEL4.1_11g015290.1	185	21,873.14	9.37	7	Type II	MIKC^C^
SmMADS41	SMEL4.1_11g015300.1	214	24,679.20	8.84	8	Type II	MIKC^C^
SmMADS42	SMEL4.1_12g007250.1	229	26,734.45	9.25	7	Type II	MIKC^C^
SmMADS43	SMEL4.1_12g007390.1	111	12,556.75	9.25	3	Type II	MIKC^C^
SmMADS44	SMEL4.1_03g026850.1	255	29,039.15	8.96	7	Type II	MIKC*
SmMADS45	SMEL4.1_04g015770.1	53	6018.15	10.10	2	Type II	MIKC*
SmMADS46	SMEL4.1_04g019230.1	312	35,774.12	5.30	8	Type II	MIKC*
SmMADS47	SMEL4.1_05g001970.1	359	41,450.34	6.77	10	Type II	MIKC*
SmMADS48	SMEL4.1_06g013880.1	138	15,508.61	5.41	5	Type II	MIKC*
SmMADS49	SMEL4.1_06g013910.1	220	24,584.16	8.57	7	Type II	MIKC*
SmMADS50	SMEL4.1_07g017280.1	197	21,873.74	5.82	8	Type II	MIKC*
SmMADS51	SMEL4.1_07g017290.1	197	21,755.52	5.09	8	Type II	MIKC*
SmMADS52	SMEL4.1_09g016090.1	77	8866.39	9.00	2	Type II	MIKC*
SmMADS53	SMEL4.1_09g016160.1	121	13,988.34	9.42	3	Type II	MIKC*
SmMADS54	SMEL4.1_09g016200.1	82	9597.44	9.47	2	Type II	MIKC*
SmMADS55	SMEL4.1_11g007660.1	58	6872.10	9.78	1	Type II	MIKC*
SmMADS56	SMEL4.1_11g007670.1	103	11,804.79	9.51	2	Type II	MIKC*
SmMADS57	SMEL4.1_11g007680.1	82	9657.53	9.89	2	Type II	MIKC*
SmMADS58	SMEL4.1_12g008860.1	591	65,950.08	4.54	11	Type II	MIKC*
SmMADS59	SMEL4.1_01g007470.1	208	23,518.86	9.94	1	Type I	Mα
SmMADS60	SMEL4.1_01g007480.1	333	37,709.97	9.10	5	Type I	Mα
SmMADS61	SMEL4.1_01g007490.1	208	23,394.70	10.00	1	Type I	Mα
SmMADS62	SMEL4.1_01g007510.1	221	25,061.62	9.37	1	Type I	Mα
SmMADS63	SMEL4.1_01g007520.1	234	26,183.44	6.76	1	Type I	Mα
SmMADS64	SMEL4.1_01g011870.1	199	21,962.69	7.69	1	Type I	Mα
SmMADS65	SMEL4.1_01g011880.1	157	17,748.33	9.87	1	Type I	Mα
SmMADS66	SMEL4.1_01g011890.1	107	11,605.35	9.94	2	Type I	Mα
SmMADS67	SMEL4.1_01g015320.1	197	22,125.41	8.88	2	Type I	Mα
SmMADS68	SMEL4.1_02g004220.1	82	9327.69	9.81	1	Type I	Mα
SmMADS69	SMEL4.1_02g004750.1	88	9891.25	8.74	1	Type I	Mα
SmMADS70	SMEL4.1_02g009330.1	163	18,034.52	6.74	1	Type I	Mα
SmMADS71	SMEL4.1_03g000810.1	177	20,440.99	6.76	1	Type I	Mα
SmMADS72	SMEL4.1_03g030700.1	176	20,252.33	8.78	1	Type I	Mα
SmMADS73	SMEL4.1_03g030950.1	177	20,399.58	8.45	1	Type I	Mα
SmMADS74	SMEL4.1_04g009890.1	131	15,092.25	8.57	2	Type I	Mα
SmMADS75	SMEL4.1_04g013110.1	213	24,212.58	7.53	1	Type I	Mα
SmMADS76	SMEL4.1_05g006350.1	196	21,900.60	5.10	1	Type I	Mα
SmMADS77	SMEL4.1_06g017190.1	160	18,834.31	9.25	1	Type I	Mα
SmMADS78	SMEL4.1_06g017740.1	195	21,797.04	9.34	1	Type I	Mα
SmMADS79	SMEL4.1_06g017750.1	221	24,990.43	9.06	1	Type I	Mα
SmMADS80	SMEL4.1_07g002870.1	154	17,280.80	9.60	1	Type I	Mα
SmMADS81	SMEL4.1_07g004050.1	177	20,399.58	8.45	1	Type I	Mα
SmMADS82	SMEL4.1_07g005030.1	82	9232.37	6.13	1	Type I	Mα
SmMADS83	SMEL4.1_07g005100.1	98	11,440.09	9.19	1	Type I	Mα
SmMADS84	SMEL4.1_07g005320.1	136	15,978.29	7.75	1	Type I	Mα
SmMADS85	SMEL4.1_08g005230.1	194	22,151.55	9.26	1	Type I	Mα
SmMADS86	SMEL4.1_08g014240.1	183	21,181.86	5.90	1	Type I	Mα
SmMADS87	SMEL4.1_09g003500.1	158	18,165.39	5.77	1	Type I	Mα
SmMADS88	SMEL4.1_09g006790.1	485	53,474.31	9.86	11	Type I	Mα
SmMADS89	SMEL4.1_09g019190.1	135	15,697.91	9.42	2	Type I	Mα
SmMADS90	SMEL4.1_10g002530.1	166	18,817.01	4.69	1	Type I	Mα
SmMADS91	SMEL4.1_10g002560.1	157	18,298.85	5.81	1	Type I	Mα
SmMADS92	SMEL4.1_10g002570.1	172	19,826.53	8.92	1	Type I	Mα
SmMADS93	SMEL4.1_10g018710.1	154	17,739.07	5.37	2	Type I	Mα
SmMADS94	SMEL4.1_10g018900.1	144	16,898.56	9.62	1	Type I	Mα
SmMADS95	SMEL4.1_11g020700.1	162	18,672.25	6.32	1	Type I	Mα
SmMADS96	SMEL4.1_11g022720.1	159	18,373.66	5.71	1	Type I	Mα
SmMADS97	SMEL4.1_12g006270.1	88	10,060.66	9.95	1	Type I	Mα
SmMADS98	SMEL4.1_12g017590.1	216	23,832.56	5.33	1	Type I	Mα
SmMADS99	SMEL4.1_12g017640.1	176	19,816.48	5.57	1	Type I	Mα
SmMADS100	SMEL4.1_01g000700.1	320	37,011.97	6.85	1	Type I	Mβ
SmMADS101	SMEL4.1_01g000740.1	312	35,650.71	6.16	1	Type I	Mβ
SmMADS102	SMEL4.1_01g000760.1	308	35,469.51	7.60	1	Type I	Mβ
SmMADS103	SMEL4.1_01g001040.1	129	14,916.10	8.96	1	Type I	Mβ
SmMADS104	SMEL4.1_03g020940.1	329	37,220.89	5.01	1	Type I	Mβ
SmMADS105	SMEL4.1_12g006420.1	296	33,732.90	4.72	1	Type I	Mβ
SmMADS106	SMEL4.1_12g006460.1	315	35,613.13	5.00	1	Type I	Mβ
SmMADS107	SMEL4.1_01g002320.1	239	27,173.99	9.18	1	Type I	Mγ
SmMADS108	SMEL4.1_01g006240.1	397	44,217.68	5.66	1	Type I	Mγ
SmMADS109	SMEL4.1_01g020650.1	318	35,923.03	7.92	1	Type I	Mγ
SmMADS110	SMEL4.1_03g004740.1	331	36,957.31	5.93	1	Type I	Mγ
SmMADS111	SMEL4.1_03g004770.1	243	28,181.97	6.24	1	Type I	Mγ
SmMADS112	SMEL4.1_03g004780.1	231	26,834.53	6.35	1	Type I	Mγ
SmMADS113	SMEL4.1_03g010570.1	180	20,547.28	6.75	1	Type I	Mγ
SmMADS114	SMEL4.1_04g005570.1	174	19,849.64	8.56	1	Type I	Mγ
SmMADS115	SMEL4.1_10g005130.1	164	19,207.47	9.28	1	Type I	Mγ
SmMADS116	SMEL4.1_10g006300.1	232	26,200.63	6.37	1	Type I	Mγ
SmMADS117	SMEL4.1_11g015800.1	397	44,978.00	5.89	1	Type I	Mγ
SmMADS118	SMEL4.1_11g016930.1	399	45,259.92	5.60	1	Type I	Mγ
SmMADS119	SMEL4.1_11g017020.1	245	28,156.78	9.01	1	Type I	Mγ
SmMADS120	SMEL4.1_11g017890.1	137	15,902.54	9.23	1	Type I	Mγ

**Table A2:** List of primers used for qRT-PCR in this study.

Primers	Sequence(5′—3′)
qPCR_SmMADS3_F	GACACCGTCTGACCAGGC
qPCR_SmMADS3_R	TAGCGGCGCTATCTCCGT
qPCR_SmMADS28_F	TCGTCCTCACGCTCAACG
qPCR_SmMADS28_R	AGGAAATCCGGTGTGGCG
qPCR_SmMADS30_F	CCCACCCAACTTTTTACCCG
qPCR_SmMADS30_R	AGACGGACTGAGAGAGCACT
qPCR_SmMADS41_F	AGACCTCGAACCCGTCCA
qPCR_SmMADS41_R	CCGATCGATGTTCGCCGA
qPCR_SmMADS75_F	ACGGGTGCTGAAATCGCT
qPCR_SmMADS75_R	ACTGCGTCAGGGCTAGGA
qPCR_SmMADS103_F	GCCATGGCGGAACAAGATC
qPCR_SmMADS103_R	TTCCCCATGAGGACCGGT
